# Asymptotic behavior of Laplacian-energy-like invariant of the 3.6.24 lattice with various boundary conditions

**DOI:** 10.1186/s40064-016-3028-1

**Published:** 2016-08-24

**Authors:** Jia-Bao Liu, Jinde Cao, Tasawar Hayat, Fuad E. Alsaadi

**Affiliations:** 1 School of Mathematics and Physics, Anhui Jianzhu University, Hefei, 230601 People’s Republic of China; 2 Department of Mathematics, and Research Center for Complex Systems and Network Sciences, Southeast University, Nanjing, 210096 People’s Republic of China; 3 Nonlinear Analysis and Applied Mathematics (NAAM) Research Group, Department of Mathematics, Faculty of Science, King Abdulaziz University, Jeddah, 21589 Saudi Arabia

**Keywords:** Lattice, Toroidal lattice, Laplacian-energy-like invariant, Laplacian spectrum

## Abstract

Let *G* be a connected graph of order *n* with Laplacian eigenvalues $\mu _1(G)\ge \mu _2(G)\ge \cdots \ge \mu _n(G)=0$. The Laplacian-energy-like invariant of *G*, is defined as ${\mathscr{L}}{\mathscr{E}}{\mathscr{L}}(G)=\sum _{i=1}^{n-1}\sqrt{\mu _i}$. In this paper, we investigate the asymptotic behavior of the 3.6.24 lattice in terms of Laplacian-energy-like invariant as *m*, *n* approach infinity. Additionally, we derive that $M ^t(n,m)$, $M ^c(n,m)$ and $M ^f(n,m)$ have the same asymptotic Laplacian-energy-like invariants.

## Background

Throughout this paper, only undirected and simple connected graphs are considered. Let *G* be a simple graph with *n* vertices. The adjacency matrix $A=(a_{ij})$ of *G* is a (0, 1)-square matrix of order *n* whose (*i*, *j*)-entry is equal to 1 if $v_i$ is adjacent to $v_j$ and equal to 0, otherwise. Let $D(G)=diag(d_1, d_2, \ldots , d_n)$ be the diagonal matrix associated to *G*, where $d_i$ is the degree of vertex $v_i$. The matrix $L(G)=D(G)-A(G)$ is called Laplacian matrix of *G*. Let *M* be a matrix representation of a graph *G*. For a graph *G*, let $M=M(G)$ be a corresponding graph matrix definned in a prescribed way. The M-polynomial of *G* is defined as $\phi _M(G; \lambda )= det(\lambda I - M)$, where *I* is the identity matrix. The M-eigenvalues of *G* are the eigenvalues of *M* together with their multiplicities. The *M*-spectrum of *G* is the multiset of M-eigenvalues of *G*. In the case of the adjacency matrix (resp. Laplacian matrix), we simply refer to the A-eigenvalues (resp. L-eigenvalues) and A-spectrum (resp. L-spectrum) as the eigenvalues (resp. L-eigenvalues) of *G*. We denote the eigenvalues of *A*(*G*), and *L*(*G*) by $\lambda _1(G)\ge \lambda _2(G)\ge \cdots \ge \lambda _n(G)$, and $\mu _1(G)\ge \mu _2(G)\ge \cdots \ge \mu _n(G)=0,$ respectively. Details on its theory can be found in recent papers (Wang 2014; Liu et al. 2014a, 2016c; Gao et al. 2012; Mohar and Alavi 1991; Liu and Pan 2015a) and the references cited therein.

For the connected graph *G*, the Laplacian-energy-like invariant of *G* (Liu and Liu 2008), is defined as ${\mathscr{L}}{\mathscr{E}}{\mathscr{L}}(G)=\sum _{i=1}^{n-1}\sqrt{\mu _i}.$ A general problem of interest in physics, chemistry and mathematics is the calculation of the Laplacian-energy-like invariant of graphs (Wang 2014; Liu et al. 2011), which has now become a popular topic of research. For more work on ${\mathscr{L}}{\mathscr{E}}{\mathscr{L}}(G)$, the readers are referred to the most recent papers (Liu and Pan 2015b; Liu et al. 2015, [Bibr CR10][Bibr CR9], ; Das and Gutman 2014).

Historically in lattice statistics, the hexagonal lattice, 3.12.12 lattice and 3.6.24 lattice have attracted the most attention (Liu and Yan 2013; Ye 2011b; Zhang 2013). Some topological indices of graphs were studied in Li et al. (2015), Yan and Zhang (2009), Ye (2011a), Liu et al. (2014b, 2016d) and Liu and Pan (2016). In fact, Liu et al. have already studied the asymptotic incidence energy (Liu and Pan 2015a) and the Laplacian-energy-like invariant of lattices (Liu et al. 2015).

It is an interesting problem to study the various energies of some lattices with various boundary conditions. W. Wang considered the behavior of Laplacian-energy-like invariant of some graphs in Wang (2014). In present paper, we derive the the Laplacian-energy-like invariant of 3.6.24 lattice via the graph spectrum of the line graph of the subdivision graph of a graph *G* with the help of computer calculation, which is different from the approach of Wang (2014). Yan et al. investigated the asymptotic behavior of some indices of iterated line graphs of regular graphs in Liu et al. (2016c). Motivated by the above results, in this paper we consider the problem of computations of the ${\mathscr{L}}{\mathscr{E}}{\mathscr{L}}(G)$ of the 3.6.24 lattice with various boundary conditions.

## Preliminaries

We first recall some underlying definitions and lemmas in graph theory.

### Some definitions and lemmas

The subdivision graph *s*(*G*) of a graph *G* is obtained from *G* by deleting every edge *uv* of *G* and replacing it by a vertex *w* of degree 2 that is joined to *u* and *v* (see p. 151 of Chartrand and Zhang 2004).

The line graph of a graph *G*, denoted by *l*(*G*), is the graph whose vertices correspond to the edges of *G* with two vertices of *l*(*G*) being adjacent if and only if the corresponding edges in *G* share a common vertex (Klein and Yi 2012).

#### **Lemma 1**

(Gao et al. 2012) *LetGbe an r-regular connected graph withnvertices andmedges, then*

$$\begin{aligned} \phi _L\Big (l(G);x\Big ) & = (x-2r)^{m-n}\phi _L(G;x),\\ \phi _L\Big (s(G);x\Big ) & = (-1)^m(2-x)^{m-n}\phi _L\Big (G;x(r+2-x)\Big ), \end{aligned}$$

*where*$\phi _L\Big (l(G);x\Big )$*and*$\phi _L\Big (s(G);x\Big )$*are the characteristic polynomial for the Laplacian matrix of graphsl*(*G*) *ands*(*G*), *respectively*.

Let a bipartite graph *G* with a bipartition $V(G)=(U,V)$ is called an (*r*, *s*)-semiregular graph if all vertices in *U* have degree *r* and all vertices in *V* have degree *s*.

#### **Lemma 2**

(Mohar and Alavi 1991) *LetGbe an* (*r*, *s*)*-semiregular connected graph withnvertices. Then*

$$\phi _L\Big (l(G);x\Big )=(-1)^n \Big (x-(r+s)\Big )^{m-n}\phi _L\Big (G;(r+s-x)\Big ),$$

*where*$\phi _L\Big (l(G);x\Big )$*is the Laplacian characteristic polynomial of the line graphl*(*G*) *andmis the number of edges ofG*.

### The 3.12.12 and 3.6.24 lattices

**Fig. 1:**
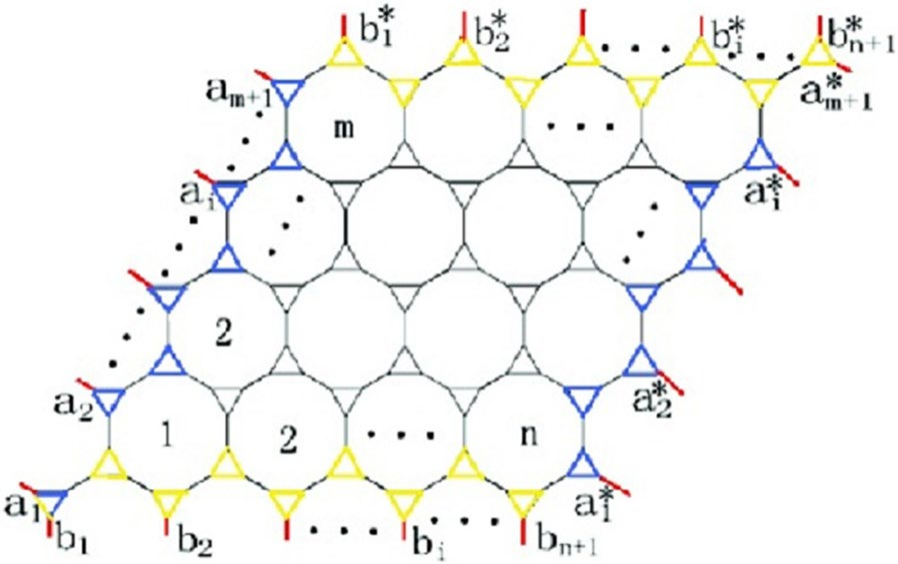
The 3.12.12 lattice $J^t(n,m)$ lattice with toroidal boundary condition

**Fig. 2:**
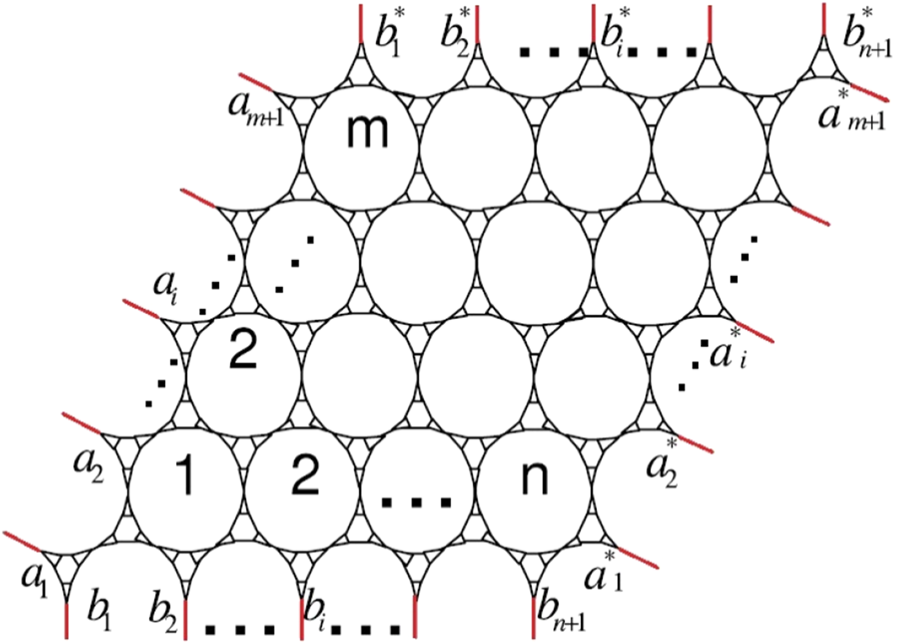
The 3.6.24 lattice $M^t(n,m)$ lattice with toroidal boundary condition

The 3.12.12 lattice with toroidal boundary condition (Liu and Yan 2013), denoted $J^t(n,m)$, is illustrated in Fig. 1. Many problems related to the 3.12.12 lattice were considered by physicists (Liu and Yan 2013; Zhang 2013; Liu et al. 2014b). The 3.6.24 lattice with toroidal boundary condition (Zhang 2013), denoted $M^t(n,m)$, is illustrated in Fig. 2.

Based on the constructions of the 3-12-12 and 3.6.24 lattices, we notice that a very important and interesting relationship between 3-12-12 lattice $J^t(n,m)$ and 3-6-24 $M^t(n,m)$ lattice. The relationship is illustrated as follows.

$$\begin{aligned}&J^t(n,m) \qquad \overrightarrow{s(J^t(n,m)) } \qquad s(J^t(n,m)) \qquad \overrightarrow{l\Big (s(J^t(n,m))\Big )}\\ &M^t(n;m) \end{aligned}$$

## Main results

In this section, we will explore the Laplacian spectrum of the 3.6.24 lattice with toroidal boundary condition. We begin with the adjacency spectrum of 3.12.12 lattice.

The following adjacency spectrum of 3.12.12 lattice is shown in Liu and Yan (2013).

### **Theorem 1**

(Liu and Yan 2013) *Let*$J^t(n,m)$*be the 3.12.12 lattice with toroidal boundary condition. Then the adjacency spectrum is*

$$\begin{aligned}&Spec_A(J^t(n,m))=\{\underbrace{-2,-2,\ldots ,-2}_{(m+1)(n+1)},\underbrace{0,0,\ldots ,0}_{(m+1)(n+1)}\}\\ & \quad \bigcup \left\{ \frac{1\pm \sqrt{13\pm 4\sqrt{3+2cos\alpha _i +2cos\beta _j+2cos\Big (\alpha _i+\beta _j\Big )} }}{2} \right\} , \end{aligned}$$

*where*$$\alpha _i=\frac{2\pi i}{m+1}, \beta _j=\frac{2\pi j}{n+1}, \,\, i=0,1,\ldots ,m;j=0,1,\ldots ,n.$$

The Laplacian spectrum of the 3.12.12 lattice with toroidal boundary condition is given by the following theorem.

### **Theorem 2**

*Let*$J^t(n,m)$*be the 3.12.12 lattice with toroidal boundary condition and*$\alpha _i=\frac{2\pi i}{m+1}, \beta _j=\frac{2\pi j}{n+1}, i=0,1,\ldots ,m;j=0,1,\ldots ,n.$*Then the Laplacian spectrum is*

$$\begin{aligned}&Spec_L(J^t(n,m))=\{\underbrace{3,3,\ldots ,3}_{(m+1)(n+1)},\underbrace{5,5,\ldots ,5}_{(m+1)(n+1)}\} \\ & \quad \bigcup \left\{ \frac{5\pm \sqrt{13\pm 4\sqrt{3+2cos\alpha _i +2cos\beta _j+2cos\Big (\alpha _i+\beta _j\Big )} }}{2} \right\} .\\ \end{aligned}$$

### *Proof*

Consider that $J^t(n,m)$ is a 3-regular graph of order *n*, then $D(G)=3I_n.$ Hence,

$$L(J^t(n,m))=3I_n-A(J^t(n,m)).$$

Define the map $\varphi (\lambda _i)=3-\lambda _i$ maps the eigenvalues of $A(J^t(n,m))$ to the eigenvalues of $L(J^t(n,m))$ and can be considered as an isomorphism of the *A*-spectrum to the corresponding the *L*-spectrum for $J^t(n,m)$. Based on the fact that *G* is an *r*-regular graph with *n* vertices and $Spec_A(G)=\{\lambda _1, \lambda _2, \ldots , \lambda _n\}.$

Then $$Spec_L(G)=\Big \{r-\lambda _1, r-\lambda _2, \ldots , r-\lambda _n\Big \}.$$$$\square$$

Next, we will deduce the Laplacian spectrum of the 3.6.24 lattice $M ^t(n,m)$.

### **Theorem 3**

*Let*$\mu _{1}\ge \mu _{2} \ldots \ge \mu _{6(m+1)(n+1)}=0$*are the Laplacian eigenvalues of the 3.12.12 lattice*$J^t(n,m)$. *Then the Laplacian spectrum of*$M ^t(n,m)$*is*

$$\begin{aligned}&Spec_L (M ^t(n,m))=\Bigg \{\underbrace{3,3,\ldots ,3}_{3(m+1)(n+1)}, \underbrace{5,5,\ldots ,5}_{3(m+1)(n+1)}\Bigg \}\\ &\quad \bigcup _{i=1}^{6(m+1)(n+1)} \left\{ \frac{5-\sqrt{25-4\mu _i} }{2} \right\} \\ &\quad \bigcup _{i=1}^{6(m+1)(n+1)} \left\{ \frac{5+\sqrt{25-4\mu _i} }{2} \right\} . \end{aligned}$$

### *Proof*

Note that $J^t (n,m)$ has $6(m + 1)(n + 1)$ vertices and $M^t(n,m)$ is the line graph of the subdivision of $J^t (n,m)$ which is a 3-regular graph. That is,

1 $$M ^t(n,m) = l(s(J^t (n,m))).$$

For the convenience of description, we suppose that $s(J^t (n,m))$ has *p* vertices and *q* edges. Obviously, $p=15(m+1)(n+1)$ and $q=18(m+1)(n+1)$, respectively. In fact, $s(J^t (n,m))$ is (2, 3)-semi-regular graphs.

By Lemma 2, suppose the graph in equality above is $s(J^t (n,m))$, then the Laplacian characteristic polynomial of $l\Big (s(J^t (n,m))\Big )$ is,

2 $$\begin{aligned} \phi _L\Big (l(s(J^t (n,m)));x\Big) & = (-1)^{p} (x-5 )^{q-p}\nonumber \\ & \times \phi _L \Big (s(J^t (n,m));(5-x)\Big ), \end{aligned}$$

By virtue of Eq. (1), one can immediately obtain that

3 $$\begin{aligned} \phi _L \Big (M ^t(n,m);x\Big) & = (-1)^p (x-5)^{q-p}\nonumber \\ & \quad \times \phi _L \Big (s(J^t (n,m));(5-x)\Big ). \end{aligned}$$

On the other hand, note that $J^t (n,m)$ has $9(m + 1)(n + 1)$ edges, it obviously follows from Lemma 1,

4 $$\begin{aligned} \phi _L \Big(s(J^t (n,m));x\Big) & = (-1)^{9(m + 1)(n + 1)}(2-x)^{3(m + 1)(n + 1)}\nonumber \\ & \quad \times \phi _L \Big(J^t (n,m);(x(5-x))\Big). \end{aligned}$$

Consider the term $\phi _L \Big (s(J^t (n,m));(5-x)\Big )$ in Eq. (3), we replace *x* with $5-x$ in Eq. (4), we have

5 $$\begin{aligned} \phi _L \Big (s(J^t (n,m));(5-x)\Big) &= (-1)^{9(m + 1)(n + 1)}(x-3)^{3(m + 1)(n + 1)}\nonumber \\ & \quad \times \phi _L\Big (J^t (n,m);(x(5-x))\Big ). \end{aligned}$$

Combing Eq. (3) with Eq. (5), $p=15(m+1)(n+1)$ and $q=18(m+1)(n+1)$, it holds

6 $$\begin{aligned} \phi _L \Big (M ^t(n,m);x\Big ) & = (-1)^{p+9(m + 1)(n + 1)} (x-5 )^{q-p}\nonumber \\ &\quad \times (x-3)^{3(m + 1)(n + 1)} \phi _L\Big (J^t (n,m);(x(5-x))\Big )\nonumber \\ & = (-1)^{24(m+1)(n+1)} \Big ((x-3) (x-5)\Big )^{3(m+1)(n+1)}\nonumber \\ & \quad \times \phi _L\Big (J^t (n,m);(x(5-x))\Big ). \end{aligned}$$

Note that the roots of $x(5-x)=\mu _{i}$ are

$$\begin{aligned} x_{1, i}=\frac{5-\sqrt{25-4\mu _i}}{2}, \quad \,\, x_{2,i}=\frac{5+\sqrt{25-4\mu _i}}{2}, \end{aligned}$$

where $\mu _{1}\ge \mu _{2} \ldots \ge \mu _{6(m+1)(n+1)}=0$ are the Laplacian eigenvalues of the 3.12.12 lattice $J^t(m,n)$.

It follows from Eq. (6) that the Laplacian spectrum of $M ^t(n,m)$ is

$$\begin{aligned}&Spec_L (M ^t(n,m))=\Bigg \{\underbrace{3,3,\ldots ,3}_{3(m+1)(n+1)}, \underbrace{5,5,\ldots ,5}_{3(m+1)(n+1)}\Bigg \}\\ &\quad \bigcup _{i=1}^{6(m+1)(n+1)} \left\{ \frac{5-\sqrt{25-4\mu _i} }{2} \right\} \bigcup _{i=1}^{6(m+1)(n+1)} \left\{ \frac{5+\sqrt{25-4\mu _i} }{2} \right\} , \end{aligned}$$

where $\mu _i$ are the Laplacian eigenvalues of the 3.12.12 lattice $J^t(n,m)$.

### **Theorem 4**

*Let*$$A=$$

$$\begin{aligned} \sqrt{5- \sqrt{15\pm 2\sqrt{13\pm 4\sqrt{3+2cos\alpha _i +2cos\beta _j+2cos\Big (\alpha _i+\beta _j\Big )}}} }, \end{aligned}$$

$$B=$$

$$\begin{aligned} \sqrt{5+ \sqrt{15\pm 2\sqrt{13\pm 4\sqrt{3+2cos\alpha _i +2cos\beta _j+2cos\Big (\alpha _i+\beta _j\Big )}}} }, \end{aligned}$$

*and*$$\alpha _i=\frac{2\pi i}{m+1}, \beta _j=\frac{2\pi j}{n+1}, i=0,1,\ldots ,m;j=0,1,\ldots ,n.$$*Then*

1. *The Laplacian-energy-like invariant of*$M^t(n,m)$* can be expressed as*
  $$\begin{aligned} {\mathscr{L}}{\mathscr{E}}{\mathscr{L}} \Big(M^t (n,m)\Big) & = 3(\sqrt{3}+\sqrt{5})(m+1)(n+1)\\ & \quad +\frac{(m+1)(n+1)}{\sqrt{2}} \Big (\sqrt{5- \sqrt{5}} +\sqrt{5- \sqrt{13}}\Big )\\ & \quad +\frac{(m+1)(n+1)}{\sqrt{2}} \Big (\sqrt{5+ \sqrt{5}}+\sqrt{5+ \sqrt{13}}\Big )\\ & \quad +\frac{1}{\sqrt{2}}\sum _{i=0}^{m}\sum _{j=0}^{n} A+\frac{1}{\sqrt{2}}\sum _{i=0}^{m}\sum _{j=0}^{n} B. \end{aligned}$$
2. $${\mathscr{L}}{\mathscr{E}}{\mathscr{L}}\Big (M^t (n,m)\Big )\approx 18.1764 (m+1)(n+1),$$* as*$$m,n\rightarrow \infty.$$

### *Proof*

Based on Theorems 2, 3 and the definition of the Laplacian-energy-like invariant, we can arrive at the statement 1 of Theorem 4.

Note that the term *A* can decompose four terms

$$A=A_1+A_2+A_3+A_4,$$

$$A_1=\sqrt{5- \sqrt{15- 2\sqrt{13- 4\sqrt{3+2cos\alpha _i +2cos\beta _j+2cos\Big (\alpha _i+\beta _j\Big )}}} },$$

$$A_2=\sqrt{5- \sqrt{15- 2\sqrt{13+ 4\sqrt{3+2cos\alpha _i +2cos\beta _j+2cos\Big (\alpha _i+\beta _j\Big )}}} },$$

$$A_3= \sqrt{5- \sqrt{15+ 2\sqrt{13- 4\sqrt{3+2cos\alpha _i +2cos\beta _j+2cos\Big (\alpha _i+\beta _j\Big )}}} },$$

$$A_4=\sqrt{5- \sqrt{15+ 2\sqrt{13+ 4\sqrt{3+2cos\alpha _i +2cos\beta _j+2cos\Big (\alpha _i+\beta _j\Big )}}} }.$$

Similarly,

$$B=B_1+B_2+B_3+B_4,$$

$$B_1=\sqrt{5+ \sqrt{15- 2\sqrt{13- 4\sqrt{3+2cos\alpha _i +2cos\beta _j+2cos\Big (\alpha _i+\beta _j\Big )}}} },$$

$$B_2=\sqrt{5+ \sqrt{15- 2\sqrt{13+ 4\sqrt{3+2cos\alpha _i +2cos\beta _j+2cos\Big (\alpha _i+\beta _j\Big )}}} },$$

$$B_3=\sqrt{5+ \sqrt{15+ 2\sqrt{13- 4\sqrt{3+2cos\alpha _i +2cos\beta _j+2cos\Big (\alpha _i+\beta _j\Big )}}} },$$

$$B_4=\sqrt{5+ \sqrt{15+ 2\sqrt{13+ 4\sqrt{3+2cos\alpha _i +2cos\beta _j+2cos\Big (\alpha _i+\beta _j\Big )}}} }.$$

Then

$$\begin{aligned} & \lim _{m\rightarrow \infty }\lim _{n\rightarrow \infty }\frac{{\mathscr{L}}{\mathscr{E}}{\mathscr{L}}\Big (M^t(m,n)\Big )}{18(m+1)(n+1)}=\frac{\sqrt{3}+\sqrt{5}}{6}\\ & \quad +\frac{\sqrt{2}}{36} \Big (\sqrt{5- \sqrt{5}}+\sqrt{5- \sqrt{13}} + \sqrt{5+ \sqrt{5}}+\sqrt{5+ \sqrt{13}}\Big ) \\ & \quad +\lim _{m\rightarrow \infty }\lim _{n\rightarrow \infty }\frac{\sqrt{2}}{36(m+1)(n+1)}\sum _{i=0}^{m}\sum _{j=0}^{n} \Big (A_1+A_2+A_3+A_4\Big )\\ & \quad +\lim _{m\rightarrow \infty }\lim _{n\rightarrow \infty }\frac{\sqrt{2}}{36(m+1)(n+1)}\sum _{i=0}^{m}\sum _{j=0}^{n} \Big (B_1+B_2+B_3+B_4\Big ). \end{aligned}$$

We consider that

$$\begin{aligned} & \lim _{m\rightarrow \infty }\lim _{n\rightarrow \infty }\frac{\sqrt{2}}{36(m+1)(n+1)}\sum _{i=0}^{m}\sum _{j=0}^{n} \Big (A_1+A_2+A_3+A_4\Big) \\ &= \frac{\sqrt{2}}{36}\cdot \frac{1}{4\pi ^2} \int _{0}^{2\pi }\int _{0}^{2\pi }A_1^{'} \,dxdy\\ & +\frac{\sqrt{2}}{36}\cdot \frac{1}{4\pi ^2}\int _{0}^{2\pi }\int _{0}^{2\pi }A_2^{'} \,dxdy\\ & \quad +\frac{\sqrt{2}}{36}\cdot \frac{1}{4\pi ^2}\int _{0}^{2\pi }\int _{0}^{2\pi }A_3^{'} dxdy\\ & \quad +\frac{\sqrt{2}}{36}\cdot \frac{1}{4\pi ^2}\int _{0}^{2\pi }\int _{0}^{2\pi } A_4^{'} \,dxdy \\ & \approx 0.0040, \end{aligned}$$

where

$$A_1^{'}= \sqrt{5- \sqrt{15- 2\sqrt{13- 4\sqrt{3+2cos x +2cos y+2cos(x+y)}}} },$$

$$A_2^{'}= \sqrt{5- \sqrt{15- 2\sqrt{13+ 4\sqrt{3+2cos x +2cos y+2cos(x+y)}}} },$$

$$A_3^{'}= \sqrt{5- \sqrt{15+2\sqrt{13- 4\sqrt{3+2cos x +2cos y+2cos(x+y)}}} },$$

$$A_4^{'}= \sqrt{5- \sqrt{15+ 2\sqrt{13+ 4\sqrt{3+2cos x +2cos y+2cos(x+y)}}} }.$$

The above numerical integration values are calculated by using the computer software Matlab.

By a complectly similar calculation with software Matlab, we can obtain that

$$\begin{aligned} & \lim _{m\rightarrow \infty }\lim _{n\rightarrow \infty }\frac{\sqrt{2}}{36(m+1)(n+1)}\sum _{i=0}^{m}\sum _{j=0}^{n} \Big (B_1+B_2+B_3+B_4\Big )\\ & \quad \approx 0.0118. \end{aligned}$$

Consequently, we have

7 $$\begin{aligned} & \lim _{m\rightarrow \infty }\lim _{n\rightarrow \infty }\frac{{\mathscr{L}}{\mathscr{E}}{\mathscr{L}}\Big (M^t(n,m)\Big )}{18(m+1)(n+1)}\nonumber \\ & \quad \approx \frac{\sqrt{3}+\sqrt{5}}{6}+\frac{\sqrt{2}}{36} \Big (\sqrt{5- \sqrt{5}} +\sqrt{5- \sqrt{13}} \nonumber \\ & \quad \quad+ \sqrt{5+ \sqrt{5}}+\sqrt{5+ \sqrt{13}}\Big ) \nonumber \\ & \quad \quad +0.0040+0.0118\nonumber \\ & \quad= 1.0098. \end{aligned}$$

The Eq. (7) implies $M^t(n,m)$ has the asymptotic Laplacian-energy-like invariant

$${\mathscr{L}}{\mathscr{E}}{\mathscr{L}}\Big(M^t (n,m)\Big)\approx 18.1764 (m+1)(n+1),$$

as $m,n\rightarrow \infty .$ The theorem thus follows. $\square$

The energy of a graph *G* with *n* vertices, denoted by ${\mathscr{E}}(G),$ is defined by

$${\mathscr{E}}(G)=\sum _{i=1}^n|\lambda _i(G)|,$$

where the $\lambda _i(G)$ are the eigenvalues of the adjacency matrix of *G*. The asymptotic energy per vertex of *G* (Yan and Zhang 2009) is defined by

$$\lim _{n\rightarrow \infty}\quad \frac{{\mathscr {E}}(G)}{\left| V(G_n)\right| }.$$

Motivated by the above results, we consider the problem of computation of the ${\mathscr{L}}{\mathscr{E}}{\mathscr{L}}(G)$ per vertex of *G* (Liu et al. 2015).

### **Theorem 5**

(Liu et al. 2015) *Let*$\{G_n\}$*be a sequence of finite simple graphs with bounded average degree such that*

$$\lim _{n\rightarrow \infty }\left| V(G_n)\right| =\infty, \quad \lim _{n\rightarrow \infty }\frac{{\mathscr{L}}{\mathscr{E}}{\mathscr{L}}(G_n)}{\left| V(G_n)\right| }=h\ne 0.$$

*Let*$\{H_n\}$*be a sequence of spanning subgraphs of*$\{G_n\}$*such that*

$$\lim _{n\rightarrow \infty }\frac{\left| v \in V(H_n):d_{H_n(v)}=d_{G_n(v)}\right| }{\left| V(G_n)\right| }=1,$$

*then*

$$\lim _{n\rightarrow \infty }\frac{{\mathscr{L}}{\mathscr{E}}{\mathscr{L}}(H_n)}{\left| V(G_n)\right| }=h.$$

*That is*, $G_n$*and*$H_n$*have the same asymptotic Laplacian-energy-like invariant*.

### *Remark 1*

Theorem 5 provides a very effective approach to handle the asymptotic the Laplacian-energy-like invariant of a graph with bounded average degree.

Based on Theorem 5, the following result is straightforward.

### **Theorem 6**

*Let*$M ^t(n,m)$*(resp. *$M ^c(n,m)$, $M ^f(n,m)$) *be the toroidal (resp. cylindrical, free) boundary condition of the 3.6.24 lattice. Then*

$$\begin{aligned}&{\mathscr{L}}{\mathscr{E}}{\mathscr{L}}\Big (M^t (n,m)\Big )\\ &\quad ={\mathscr{L}}{\mathscr{E}}{\mathscr{L}}\Big (M^c(n,m)\Big ) \\ & \quad ={\mathscr{L}}{\mathscr{E}}{\mathscr{L}}\Big (M^f (n,m)\Big )\\ & \quad \approx 18.1764 (m+1)(n+1). \end{aligned}$$

### *Remark 2*

It follows from Theorems 5 and 6 that the growth rate of the ${\mathscr{L}}{\mathscr{E}}{\mathscr{L}}(G)$ of the 3.6.24 lattice $M ^t(n,m)$ (resp. $M ^c(n,m)$, $M ^f(n,m)$) with toroidal (resp. cylindrical, free) boundary condition is only dependent on the number of vertices of it.

## Conclusions

In this paper, we deduced the formulae expressing the Laplacian-energy-like invariant of the 3.6.24 lattice with various boundary conditions. Moreover, we obtained the explicit asymptotic values of the Laplacian-energy-like invariant by utilizing the analysis methods with the help of software Matlab calculation. In addition, we showed that their growth rates are independent of the structure of *M* (*n*, *m*) and only dependent on the number of vertices of *M* (*n*, *m*). These and some other related issues are very good topics on lattices, which deserves further exploration.
